# Function and Evolution of the Loop Extrusion Machinery in Animals

**DOI:** 10.3390/ijms24055017

**Published:** 2023-03-06

**Authors:** Evelyn Kabirova, Artem Nurislamov, Artem Shadskiy, Alexander Smirnov, Andrey Popov, Pavel Salnikov, Nariman Battulin, Veniamin Fishman

**Affiliations:** 1 Institute of Cytology and Genetics SB RAS, 630090 Novosibirsk, Russia; 2 Department of Natural Sciences, Novosibirsk State University, 630090 Novosibirsk, Russia; 3 Artificial Intelligence Research Institute (AIRI), 121108 Moscow, Russia

**Keywords:** loop extrusion, evolution, SMC complexes, cohesion, gene regulation

## Abstract

Structural maintenance of chromosomes (SMC) complexes are essential proteins found in genomes of all cellular organisms. Essential functions of these proteins, such as mitotic chromosome formation and sister chromatid cohesion, were discovered a long time ago. Recent advances in chromatin biology showed that SMC proteins are involved in many other genomic processes, acting as active motors extruding DNA, which leads to the formation of chromatin loops. Some loops formed by SMC proteins are highly cell type and developmental stage specific, such as SMC-mediated DNA loops required for VDJ recombination in B-cell progenitors, or dosage compensation in *Caenorhabditis elegans* and X-chromosome inactivation in mice. In this review, we focus on the extrusion-based mechanisms that are common for multiple cell types and species. We will first describe an anatomy of SMC complexes and their accessory proteins. Next, we provide biochemical details of the extrusion process. We follow this by the sections describing the role of SMC complexes in gene regulation, DNA repair, and chromatin topology.

## 1. The Origin and Evolution of the Loop Extrusion Machinery

**Figure 1:**
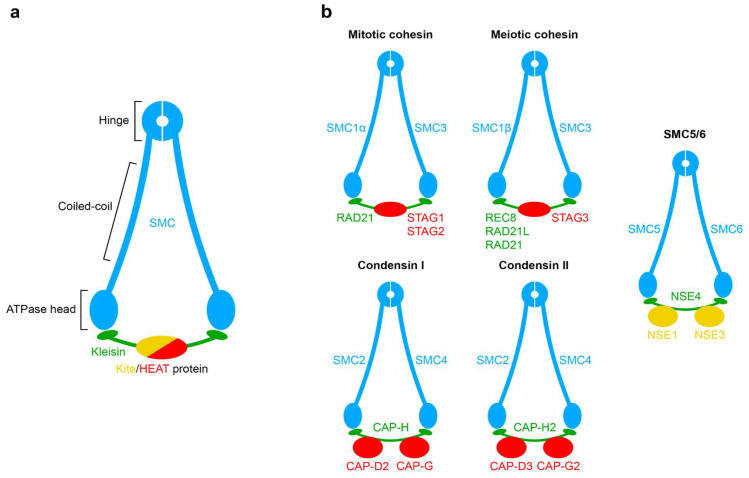
Basic structure of SMC complexes. (**a**) Main parts of SMC complex; (**b**) eukaryotic SMC–kleisin complexes (blue and green) with partner proteins (red and yellow).

Loop extrusion (LE) machinery consists of various proteins with different time origins, however, the evolutionarily conserved core of all extrusion complexes is formed by SMC proteins (Figure 1a)—ATP-dependent protein machines which are present in all species from bacteria and archaea to humans [1]. A bacterial SMC complex consists of two special antiparallel coiled-coil proteins (k-Smc and v-Smc) that have an ABC-type ATPase domain at the end (so-called ‘head’) [2]. On the opposite side, they form a homodimeric structure—a ‘hinge’ [3]. In addition, ScpAB subunits link the head of one protein to the coiled-coil of the other [4]. There is also an analogue of this SMC complex—MukBEF—that is present mostly in γ-proteobacteria [5].

The SMC complexes in prokaryotes play an important role in the process of chromosome segregation [6,7].

**Figure 2:**
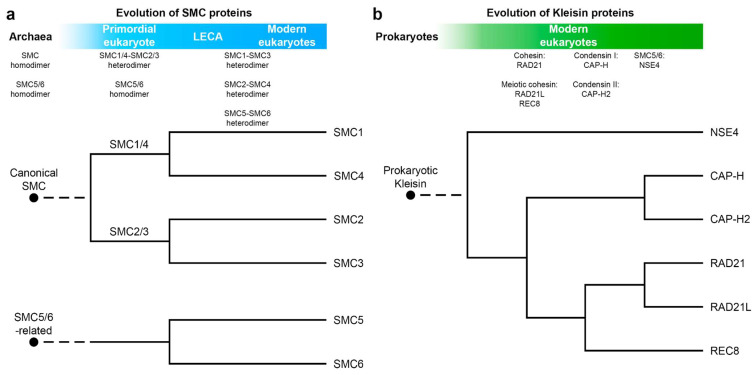
Origins of eukaryotic SMC and kleisin proteins. (**a**) A hypothetical model for evolution of SMC proteins in eukaryotes; (**b**) divergence of kleisins in modern eukaryotes.

In eukaryotes, SMC proteins are found in cohesin, condensins, and SMC5/6 complexes (Figure 1b). It is suggested that divergence of SMC in archaea resulted in canonical SMC and SMC5/6-related protein. Duplications of ancestral eukaryotic canonical SMC gave rise to SMC1/4 and SMC2/3 families. Further duplications and functional diversification led to the formation of modern SMC1–6 proteins [1,8] (Figure 2a). In contrast to prokaryotes, eukaryotic SMCs act as heterodimers: SMC1–SMC3 in cohesin, SMC2–SMC4 in condensins, and SMC5–SMC6 in SMC5/6 DNA repair complexes.

The kleisin superfamily also originates from prokaryotes (Figure 2b)**.** Prokaryotes possess ScpA/MukF as a kleisin protein which acts as a bridge between heads of homodimeric SMC/MukB complexes [9]. Depending on the type of complex, different kleisins bridge heterodimeric SMC complexes in eukaryotes: RAD21/SCC1 in cohesins [10,11] (as well as RAD21L and REC8 meiotic variants [12,13,14]), CAP-H/CAP-H2 in condensins [15], and NSE4 in SMC5/6 [16]. SMC–kleisin complexes across the tree of life have various binding partners with different functions.

Most prokaryotic SMC–kleisin partner proteins belong to the kleisin interacting winged-helix tandem elements (Kite) family [17]. However, not much is known about the origins of SMC–kleisin partner proteins in eukaryotes except for NSE1 and NSE3 in the SMC5/6 complex which belong to the Kite family. It is proposed that, during the evolution of SMC–kleisins in eukaryotes, Kite proteins were replaced with proteins containing HEAT repeats [18] (e.g., SCC3/SA/STAG, SCC2/NIPBL, PDS5, and WAPL in cohesin, CAP-D and CAP-G in condensins). Secondary losses of SMC–kleisin subunits and partner proteins can occur during evolution [8,19] as well as additional duplication events. For example, loss of condensin II subunits results in Rabl-like chromosome configuration [20,21]. Zebrafish have four Stag paralogues (Stag1a, Stag1b, Stag2a, Stag2b) instead of two as in other vertebrate classes [22].

## 2. Structural Details of Interactions between SMC Complexes and DNA

Both eukaryotic and prokaryotic SMC complexes can either hold DNA molecules or actively translocate DNA through the process defined as loop extrusion. We describe the biochemical details of interaction between SMC complexes and DNA based on the data available for human SMC complexes.

Current experimental and cryo-EM data show that human cohesin forms the SMC1–SMC3 (orthologs of prokaryotic k-Smc and v-Smc) heterodimer that interacts with RAD21, the kleisin subunit protein, through the winged-helix domain (WHD) in SMC1 and N-terminal helical domain (NHD) in SMC3 [23,24]. Kleisin thus connects with its N-terminus to the head domain (HD) of SMC3 and with the C-terminus to the HD of SMC1, forming a small ring.

The NIPBL–MAU2 complex includes two proteins and is responsible for loading SMC complexes on chromosomes (so-called ‘loading complex’). It forms a ‘U’-shaped form that contains 24 HEAT repeats (R1–R24) and a helical insert domain (HID). The NIPBL protein binds to the RAD21 through both electrostatic and hydrophobic interactions through amino acid residues 154–171 that are related to amino acid residues 126–230 of Scc1 in yeast cells [25].

There are three regions through which NIPBL protein binds with the SMC1–SMC3 complex. The first one is formed by SMC1 and SMC3 HDs and R18–R21 of NIPBL that enhances the heterodimeric interactions between HDs of SMC1 and SMC3. The second includes the interaction between the SMC1 coiled-coil and C-terminal region of NIPBL through mostly hydrogen bonding and hydrophobic interactions. The third region is between the SMC3 joint, the R2–R6 of NIPBL, and NHD of RAD21 and occurs due to electrostatic and hydrophobic interactions. Generally, NIPBL is essential for SMC1 and SMC3 stabilizing of HD-engaged SMC1–SMC3 dimerization and for DNA entrapment [24].

STAG1, the protein that is linked to the kleisin, also adopts a ‘U’-shaped form and there are two different regions where NIPBL and STAG1 interact. The major one consists of R8–R10 of STAG1 and R1–R5 of NIPBL. The smaller region includes interaction between R1 and R2 of STAG1 and R13 of NIPBL. Moreover, STAG1 connects directly to the SMC1–SMC3 complex through the SMC1 hinge and R8–R9 of STAG1 [24]. There are also interactions between RAD21 and STAG1: STAG1 binds to RAD21 through 321–345 residues of the latter in the N-terminal region and through 356–395 residues in the central region by R9–R14 [26].

DNA binds to all the subunits of the SMC1–SMC3 with NIPBL/STAG1 complex. The R-loop and succeeding β-sheet in the N-terminal region of both SMC1 and SMC3 are the major DNA binding sites. The RAD21, NIPBL, and STAG1 bind to the DNA molecule. However, the latter has the smaller binding region in the N-terminal part of the helix in R8 and an extended loop following that helix [24].

The mechanism of DNA entering into the cohesin ring remains similar in yeast and human cells. The HDs of SMC1 and SMC3 stay engaged when they are bound with ATP, forming a ring-shaped structure. DNA binds at the top of the SMC complex HDs and coiled-coils, appearing to be entrapped in the SMC ring. The ATP binding to the HDs stimulates the dissociation of the N-terminus of kleisin from SMC3—this process is called ‘N-gate opening’. Then cohesin enters ‘the gripping state’ as the N-terminus of kleisin binds again to the SMC3, thereby DNA may enter the cohesin with both of its ends or the second one may leave the ring through the opened N-gate [27].

There are three different ways DNA can enter the SMC complex. In the first one, a DNA molecule enters the ring while in the second it leaves through the opened N-gate, but still keeps being held by electrostatic interactions with the loading complex, and is moved outside by STAG1. However, the experimental data show that LE machinery proceeds either pseudo-topologically or non-topologically [28]. The first way occurs when both of the DNA molecules actually enter the SMC complex and after ATP hydrolysis one of the molecules is held by a hinge subunit and the other is entrapped in the cohesin by the kleisin protein. The second one is the best in explaining how the LE process can bypass large obstacles [29]. In it, DNA does not enter the cohesin at all and is thought to be held by electrostatic forces in the hinge subunit and the NIPBL–MAU2 complex.

## 3. The Mechanism of DNA Translocation by SMC Complexes

There are several models explaining biochemistry of the DNA translocation process. Among them, two varieties of LE can be distinguished: one posits that each motor subunit of the SMC complex translocates in opposite directions. This model is called two-sided loop extrusion. The other one, one-sided loop extrusion, suggests that a fraction of SMC complexes reel in DNA from only one side, while the other side remains anchored to its DNA loading site [30]. For instance, asymmetric (one-sided) LE can be detected for yeast [31] and *C. elegans* condensin [32]. Theoretical studies have shown that symmetric (two-sided) LE is necessary for larger vertebrate genomes [33]. Thus, for both human condensins the LE is mostly symmetric, but it is suggested that the two-sided symmetry may be a result of two asymmetric condensin complexes [34]. Another possibility is one-sided loop extrusion with frequent switches of extrusion side.

The most modern model is a Brownian ratchet model which suggests that an SMC complex having captured DNA in the ATP-bound form spatially acquires a state that is bent at the elbows, in which the hinge and STAG1 are connected and form a complex. Then, after ATP hydrolysis, STAG1 loses its connection with the NIPBL protein and moves away from the latter by Brownian motion, lengthening the resulting DNA loop. Thus, repeating cycle after cycle, the loop extrusion mechanism occurs [35].

A similar but a slightly outdated model is the ‘tethered-inchworm’ model. It suggests that the sequential binding and hydrolysis of ATP lead to the opening and closing of the SMC ring that pulls DNA [36]. In the ATP-bound form, ‘heads’ of the SMCs form a bridge that separates a whole cohesin ring into two compartments: a large ‘SMC’ and a smaller ‘kleisin’. The latter is responsible for trapping DNA [37]. Therefore, the alternation of binding ATP, hydrolysis, and release cycle leads to large-scale changes in the conformation of SMC complexes that are thought to induce a loop extrusion.

An alternative DNA segment capture model comes from the working features of bacterial SMC complexes. The main point of this model is that the SMC complex changes from the state of two rings to the state of one upon hydrolysis of ATP. In this case, DNA first enters the small kleisin ring, and upon hydrolysis of ATP, it binds to the SMC head domains. Then, it also connects with the domains near the hinge, forming a loop [38].

In turn, a scrunching model reveals that DNA loop extrusion is a change in the conformation of the condensed complex. The transition between the open O shape and the closed B shape depends on the presence of ATP and forms a motor mechanism that transfers DNA between the globular domain and the hinge domain of the SMC complex [39]. A variant of this model, known as swing and clamp, shows that the upper spiral coils of the SMC subunits bend spontaneously, which leads to a 50 nm movement of the hinge to the heads [40].

Although the loop formation is a very dynamic process, there is a mechanism that can regulate it. This mechanism is mediated by CTCF proteins which recognize special sites in DNA by the ZF domain and bind to them [41]. It was shown that antiparallel CTCF binding sites with CTCF on them stop the loop extrusion process, thereby forming a relatively stable loop [42]. Recent studies suggest that CTCF binds to the 86-amino acid motif called the ‘conserved essential surface’ (CES) of the STAG1/2–RAD21 complex. This interaction leads to the stabilization of the loop and stops the loop extrusion process. The importance of the antiparallel direction of CTCF sites is that the N-terminus of CTCF binds to the STAG1/2–RAD21 complex [43].

One of the difficulties in establishing the loop extrusion machinery is how it is consistent with other processes occurring in the cell. In vivo, chromatin is associated with a huge amount of diverse elements, such as nucleosomes, RNA polymerases, spliceosomes, and others. At first glance, the presence of extraneous elements may interfere with loop extrusion. In fact, even DNA-bound nanoparticles up to 200 nm in size, much larger than the SMC ring size, do not interfere with the movement of DNA [29]. Moreover, these elements may be part of the loop extrusion machinery. For example, transcribing RNA polymerases can act as ‘moving barriers’ by obstructing, slowing down, or pushing loop-extruding cohesins [44]. On the other hand, specific proteins may have a previously unrecognized function as loop extrusion barriers—such as, for example, recently identified for MCM replication complexes [45].

## 4. Functions of the Loop Extrusion Machinery

**Figure 3:**
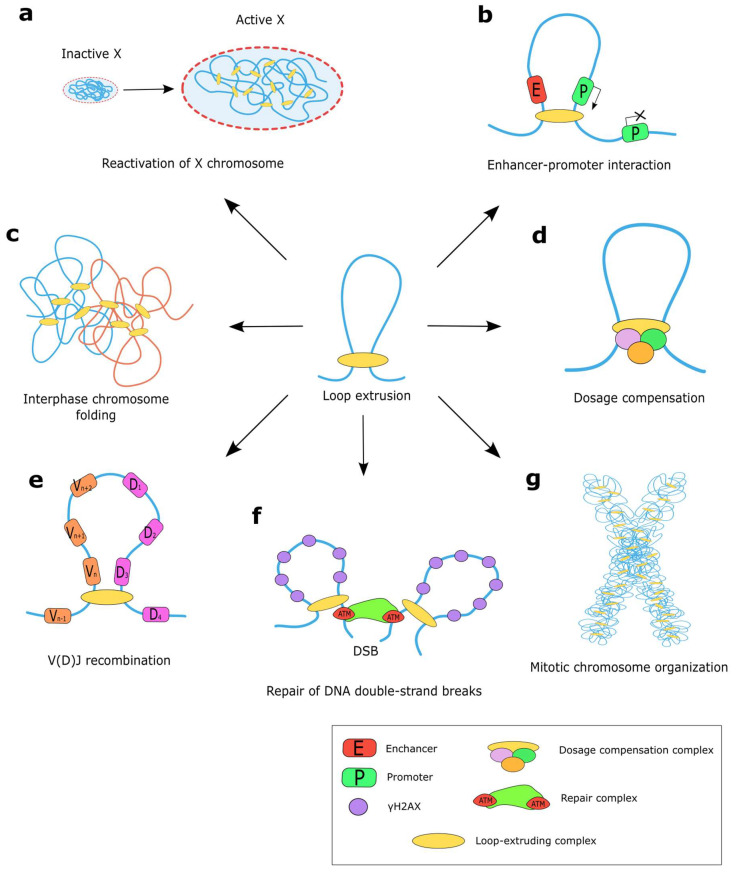
The functions of SMC complex. (**a**) The cohesin complex member SMC1a is hypothesized to provide reactivation of the inactive X chromosome of mice [50]. (**b**) Loop extrusion can prevent enhancer–promoter interactions by isolating them from one another or can facilitate enhancer–promoter interactions by converging them. (**c**) Formation of loops by cohesin is essential for establishing chromosome territories and suppressing interchromosome entanglements in vertebrate cells. (**d**) Loop extrusion by specialized condensin complex participates in dosage compensation of *C. elegans* [32]. (**e**) Loop extrusion activity mediates bringing DNA segments together during V(D)J recombination. (**f**) Cohesin-mediated loop extrusion on both sides of the double-strand breaks allows ATM to phosphorylate H2AX containing nucleosomes signaling for the repair system [51]. (**g**) Condensin complexes are a crucial factor determining compaction of chromosomes during mitosis [52].

SMC-required proteins are involved in many genomic processes (Figure 3a–g), acting as active motors extruding DNA, which leads to the formation of chromatin loops [46,47]. Some loops formed by SMC proteins are highly cell type and developmental stage specific, such as SMC-mediated DNA loops for VDJ recombination in B-cell progenitors [48], or dosage compensation in *C. elegans* [49] and X-chromosome inactivation in mice [50]. Other functions, such as regulation of gene expression, chromatin compaction, and assistance of the DNA repair machinery, are more universal. Below, we describe the role of SMC complexes in these processes.

### 4.1. Loop Extrusion for Gene Regulation

In vertebrates, loop extrusion drives an establishment of chromatin structures called topologically associating domains (TADs). TADs are continuous regions of genome, typically several hundreds of kilobases in length [53], enriched for self-interactions but insulated from their genomic neighborhoods [54]. Although TADs are observed both in vertebrates and invertebrates, mechanisms underlying TAD formation in these taxa are different (see review by V. Corces and colleagues [55]). In vertebrates, cohesin complexes translocate DNA within TADs, increasing the number of intra-TAD contacts and stalling when encountering TAD boundaries occupied by CTCF, resulting in decreased probability of inter-TAD contacts.

Loop extrusion folding chromatin into TADs is a dynamic process of switching between unlooped and looped states with different duration and frequency. In a 500 kb TAD, encompassing a silent gene *Fbn2* in mESCs, both of these states exist only for a small portion of the time (fully looped for 3% of the time, unlooped for 6%), while a partially extruded state is predominant [56]. CTCF-anchored loops were revealed to generally last about 10 min [57]. It might seem too short, but cohesin and CTCF constrain a chaotic global chromatin movement, ensuring an increase in specific cis interactions within a TAD [57]. Thus, spatial chromosome architecture forms a complex contact pattern for precise gene regulation.

Although the difference between the frequency of intra-TAD and inter-TAD chromatin contacts is relatively small (only ~2–3 times) [54,58], CTCF-associated TAD boundaries are typically conserved across cell types and syntenic loci of close species [54,59]. In line with this, the mutations causing TAD fusion are under negative selection in the population [60]. Nonetheless, a change in TAD structure can form a new pattern of gene expression presenting a material for natural selection. An example of a TAD disruption, which resulted in an evolutionary advantage, is moles’ adaptation to an underground lifestyle. An aberrant expression of the protesticular growth factor gene *Fgf9* induced masculinization of female oogenesis and occurred in moles due to a TAD rearrangement at the locus [61]. Thus, TADs’ role in establishing and maintaining gene regulation patterns is one of the most intriguing questions in chromatin biology.

TADs are believed to participate in gene regulation by control of spatial contact rates between regulatory elements. The function of TADs may be assumed to bring an enhancer and promoter in proximity, as well as isolating the effect of an enhancer on non-target genes. Exploring TADs’ role in gene regulation is based on the notion that since the CTCF–cohesin complex establishes a TAD structure, removing CTCF binding sites (CBSs) at the border would cause fusion of adjacent TADs, henceforth resulting in an ectopic activation of genes, or enhancer hijacking [62,63].

Several studies starting from an observation of TAD disruptions in patients were able to prove that such mutations can cause a pathogenic phenotype with a proto-oncogene activation [64,65,66]. It appears that many boundary CBSs are mutated near proto-oncogenes [67,68,69], suggesting an important role of TAD integrity. However, only a small portion of these mutations cause an increase in expression in nearby genes [70,71]. The importance of TADs is further challenged by mouse models of genome-edited TAD mutations, which have shown different effects on gene regulation, from absent [72] or moderate [72,73,74] expression changes to dramatic phenotypic manifestations [64].

The genome-wide TAD disruption by depletion of CTCF or cohesin does not induce dramatic expression alteration (expression of only 1.2% genes changed more than 75%) [75]. Such unexpectedly moderate depletion consequences contradict the belief that the spatial genome architecture has an essential role for gene expression regulation. It is suggested that CBS-demarcated TADs are conservative elements that shape the invariant spatial backbone within which tissue-specific spatial elements are formed by other architectural proteins, such as the Mediator complex [76] and PRCs [77].

Altogether, these seemingly inconsistent results indicate that TADs’ role in genetic regulation is highly dependent on the context of an individual locus. Below, we discuss the features which potentially influence the variety of TADs’ regulatory functions: TAD border characteristics, the distance and compatibility between regulatory elements, an epigenetic landscape (especially an active transcription).

**Figure 4:**
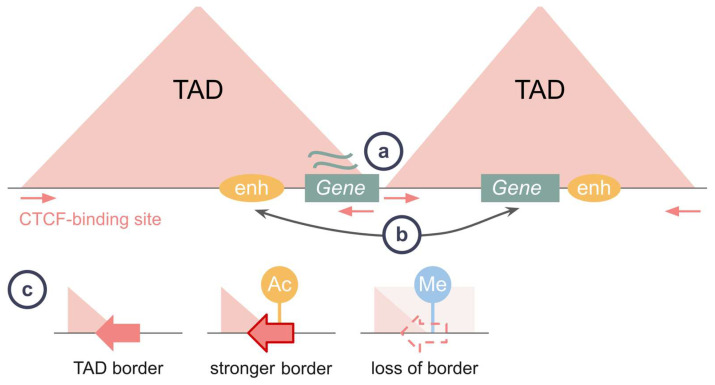
What contributes to the gene regulation by TADs? (**a**) CTCF binding sites at a TAD border provide robustness of the regulation within a TAD. (**b**) A decreased distance between regulatory elements may cause a rearrangement of spatial chromatin contacts, ensuring a newly established regulatory pattern. (**c**) An epigenetic landscape of an intra-TAD region or a TAD border maintains appropriate gene regulation. An active transcription of a border region ensures robustness of a TAD, while hypermethylation counteracts CTCF binding at a TAD border, which may alter gene expression.

CTCF sites at the TAD border are crucial to form a TAD structure. The TAD boundary typically contains multiple CBSs forming a cluster. Though individual CBSs can have different insulating strengths, their contribution to maintenance of the TAD border is not strictly correlated with the level of CTCF occupancy [78]. Moreover, CBSs might be dispensable to maintain a border [79,80]. These features of the border CBSs provide robustness of TAD integrity, ensuring an adequate gene regulation pattern (Figure 4a).

A difference between designs of experiments concerning the effect of TAD fusion on gene expression could contribute to the assumption about an inconsistency of CBS function. For instance, there is a difference in the sizes of deletions chosen to obtain TAD fusion, which vary from 10 bp [66,81] and several kilobases [72,74,82] to a couple of megabases [64]. An overall tendency is that larger deletions are more likely to result in an ectopic gene activation [80,82]. The decrease in genomic distance between regulatory elements alone may contribute to an enhancer hijacking [83] and a rearrangement of spatial chromatin contacts, inducing a regulatory landscape rewiring. Thus, genomic distance may contribute to an effect of TAD fusion on gene regulation in addition to a TAD functional role (Figure 4b).

Another subject to consider is that the larger alterations could also include a portion of regulatory regions and epigenetic landscapes, which may affect gene regulation in addition to an absence of border CBSs. The genetic and epigenetic context of a TAD may alter enhancer basal activity, as was revealed for the *HoxD* locus, which is divided between two TADs in accordance with *HoxD* gene activity during proximal or distal limb development [84]. Transferring a distal limb enhancer into the proximal limb TAD resulted in silencing of its initial activity and inducing the proximal activity, while removing a portion of the TAD context rescued the initial activity. Functionality of a TAD border depends on its epigenetic features. Hypermethylation counteracts CTCF binding, which can cause TAD disruption and an abnormal gene activation, as was shown for activation of the oncogene *PDGFRa* in *IDH* mutant gliomas [66]. Transcription of a TAD border, whether it is genic or non-genic, is argued to cause an increase in CTCF occupancy, therefore increasing the border robustness [85,86]. Moreover, transcriptional molecular machinery can directly interfere with cohesin shifting a TAD border, which provides an additional mechanism of regulation [44,87].

Overall, an epigenetic landscape influences TAD integrity and regulation within a TAD in various ways (Figure 4c). Motivated by this observation, several approaches for predicting TAD strengths based on other epigenetic inputs were recently developed [88]. However, none of them achieve absolute accuracy, indicating that not all epigenetic mechanisms and factors influencing TADs are discovered. In addition, a lack of systematic benchmarks between these methods complicates unbiased estimation of their efficiency [89].

Epigenetic status within TADs is of great interest in the context of evolution. Formation of a TAD has been associated with evolutionary events of integrating new genes or enhancers into existing regulatory landscapes in order to prevent their disruption [90,91].

In the context of the TADs’ conservativity, the wide variety of epigenetic influences on TADs suggests an establishment of tissue-specific features within different epigenetic landscapes. An epigenetic context could underlie a different outcome of TAD disruption depending on cell type, as was revealed for the *Kit* locus in mouse mast cells and melanocytes [92]. Comparison of the same CBS deletion in the two primary cell cultures revealed that an ectopic gene activation was able to occur only in the context of melanocytes. Thus, TADs’ participation in gene regulation could be locus and tissue specific.

Finally, regulatory elements may exhibit mutual selectivity [93,94,95]. Rules for the enhancer–promoter compatibility are still not entirely clear, so it is difficult to predict the reassociation of regulatory elements after TAD fusion.

Nonetheless, even TAD fusion does not guarantee an expression change [72,82]. There are a number of studies showing that TAD boundary inversion, duplication, or ectopic TAD boundary formation is generally more likely to affect gene regulation than TAD boundary deletion [73,96]. Therefore, it can be assumed that evolutionary forces that remove isolating boundaries between interconnected regulatory elements mainly dictate the position of TADs, and not the need to help cis elements spatially interact.

The study of the genome-wide TAD positioning patterns can help to reveal their regulatory role. Many large TADs contain only one gene, which in most cases turns out to be a key player of development, having a complex regulatory landscape [97]. The main function of these TADs, as hypothesized, is to isolate developmental genes requiring fine expression control from environmental interference.

In contrast, there is some pure evidence that CBS-mediated spatial organization can direct interactions for precise control of gene expression. The most well-known examples are alternative promoter choice, where cis-regulatory elements are recruited into spatial proximity with an alternative transcription start site, bound by architectural proteins [98]. However, most of these cases seem to be particular evolutionary events where the CBS is utilized for a specific purpose.

While loop extrusion participates in gene regulation through TAD formation, its role is not as essential as was initially presumed and depends on other factors, which were revealed to be locus and tissue specific. Conceivably, TADs’ role in uniting a regulatory landscape could be of great importance for minimizing consequences of perturbations on adjacent regions due to evolution.

### 4.2. Co-Evolution of CTCF and Cohesin Proteins in Bilateria

The studies described above show the role of CTCF-mediated TADs in the control of gene expression in vertebrates. However, in some species CTCF does not block loop extrusion [99]; moreover, multiple species lack CTCF in their genomes [100]. How and when interactions between CTCF and loop-extruding complexes evolve is one of the open questions in chromatin biology.

CTCF emerged later than cohesin—only around 500 Mya in Bilateralia [100]. CTCF contains an N-terminal domain, DNA binding domain, and C-terminal domain. The zinc-finger DNA binding domain is conserved and binds to similar DNA motifs in distant groups of bilateral animals [101,102,103]. On the other hand, the N-terminal domain is highly variable across Bilateralia [104]. The C-terminal domain contains the nuclear localization signal and phosphorylation sites [105]. Recent studies revealed that the N-terminal domain plays a key role in the binding of CTCF to cohesin, namely, the YXF motif corresponding to positions 226–228 in human CTCF [42,43]. Despite some differences in the structure of the N-terminal domains across Bilateralia, it was shown that the YXF motif, which is critical for the formation of chromatin loops by the CTCF/cohesin loop extrusion mechanism, is retained in the N-terminal domain in species where CTCF does not participate in loop formation (e.g., *Drosophila* [99,104] and *Anopheles* [21]). A mutation in this motif in *Drosophila* CTCF (dCTCF) results in the inability of CTCF to bind to Vtd–SA complexes (homologous to human RAD21–STAG1/2) in vitro [104], which is similar to the effect observed in vertebrates [42,43]. In this regard, the reason why CTCF may be involved in the formation of chromatin loops in vertebrate taxa [46,106,107,108,109] and not in other taxa is unknown. Possibly, this may be due to both differences in the structure of CTCF and in the structure of proteins included in the cohesin complex. Additionally, dCTCF lacks the KTYQR motif in the N-terminal domain which binds to PDS5A protein in humans and is conserved in jawed vertebrates [104]. CTCF is present in basal nematodes (e.g., *Trichinella spiralis*) but absent in *C. elegans* and other derived nematodes due to secondary loss [100]. CTCF in *Trichinella spiralis* consists of only 10 zinc finger domains instead of 11 and is enriched with multiple polyglutamine tracts [110] which may indicate loss of function instead of neofunctionalization. Secondary loss of CTCF also occurred in Platyhelminthes [100].

It is still unclear at which moment CTCF/cohesin loop extrusion emerged as a mechanism for formation of loops and TADs. Recent studies on *Branchiostoma* species [111] showed that domain borders are enriched with CTCF binding sites which indirectly indicates that CTCF/cohesin loop extrusion may be present not only in vertebrates but in Cephalochordata too. Further research on Cephalochordata and deuterostomes in general may reveal origins of the CTCF-mediated loop extrusion mechanism [112].

### 4.3. Loop Extrusion for DNA Repair

In this section, we focus on the role of LE in double-strand break repair (DSBR) in animals, because correct joining of the two DNA ends in a vast nuclear space is inextricably connected to DNA topology. Out of all SMC complexes, we will solely focus on cohesin here since condensin complexes do not directly bind to the DSB [113]. Moreover, knockdown of condensin I does not affect DSB repair, although the complex may have functions associated with the repair of single-stranded regions formed during base excision repair (BER) [114,115]. At the same time, condensin II is likely involved in the prevention of DNA damage, including DSBR [116,117], but the role of LE in the process is poorly understood. The third SMC complex, SMC5/6, like cohesin and condensins, is able to extrude DNA loops, however, its role in maintaining chromatin architecture is not well known [118]. Presumably, SMC5/6 have a role similar to cohesin in DSBR, since knockout of these proteins leads to a decrease in sister chromatid exchanges [119]. Experiments with inducible degradation of the SMC5/6 components with an auxin degron system [120] demonstrated that their absence in the S-phase leads to the accumulation of collapsed replication forks and R-loops. R-loops are DNA–RNA three-stranded nucleic acid structures which play an important role in regulating gene activity and maintaining genome stability. They often begin to accumulate in the genome when the double-strand break repair system is disturbed [121].

Most DSBR is performed by two competing molecular pathways: homologous recombination (HR) and non-homologous end-joining (NHEJ), of which HR is more relevant to cohesin and LE, since its activity is restricted to the S/G2-phase of the cell cycle when two chromatids are available [122]. In HR, DNA ends are sensed and bound by the MRE11–RAD50–NBS1 (MRN) complex which facilitates initial end trimming [122] and holds DNA molecules together [123]. The MRN complex also recruits the phosphoinositide 3-kinase-related protein kinase (PIKK) family ATM kinase that is critical for early response and attracting DSB-processing factors. Among these factors, cohesin is also recruited to the DSBs via NIPBL–MRN interaction [124]. ATM and SMC5/6 are also required for cohesin recruitment [51,125]. After that, the 5′–3′ end resection is carried out to generate RPA-covered single-stranded DNA. Interaction of cohesin with single-stranded DNA may serve as a regulator of local cohesin binding. After the DNA end resection, a concerted action of mediator proteins (Rad52, Rad54, BRCA2) assembles nucleoprotein filament by replacing RPA with Rad51 recombinase [126]. Nucleoprotein filament searches homologous substrates by random non-specific collisions and shuffling of double-stranded DNA until a stable D-loop and DNA synthesis are achieved [127,128]. Apparently, this process is very sensitive to the local DNA topology, as recombination frequency correlates with regions’ proximity. In most cases, a sister chromatid is used as a template for recombination [129].

NHEJ is the alternative DSBR mechanism preferentially working during G1-phase. It consists of the Ku complex, single phosphatidylinositol 3-kinase DNA-PKcs, a set of DNA-modifying enzymes (endonucleases, polymerases), and the ligase4–XRCC4 complex. NHEJ machinery binds non-resected DSB ends, prepares two ends for ligation, and joins them directly [130]. SMC complexes, including cohesin, both condensins, and SMC5/6, are not recruited to DSBs during G1-phase [125,131]. However, the presence of cohesin can interfere with the mobility of the DSB ends, thus preventing the distal ends of the DSB from being joined by NHEJ. Thus, cohesin can regulate the balance between NHEJ and HR during DSB repair [131].

Initially, cohesin was identified as an important factor required for resistance to UV and IR damage [132]. Until recently, its role in DSB repair was mainly attributed to its structural function in a sister chromatid cohesion. A detailed description can be found in a handful of comprehensive reviews [133,134]. In brief, cohesin was shown to be important for DSBR specifically in S/G2-phase, after DNA replication. Cohesin knockdown led to an increase in the frequency of deletions (i.e., the joining of two distant ends of DNA) [131]. However, this observation only applies to distant DSBs, as the absence of cohesin does not affect the repair efficiency of proximal DSBs (34 bp) [131,135]. Although cohesin is recruited early, it is not required per se for most DSBR steps, such as DNA end tethering [136], resection [137], or Rad51-dependent nucleoprotein filament formation [138]. Yet, new findings hint that cohesin affects D-loop stabilization for DNA synthesis [139], and remodels local chromatin, affecting DNA damage response (DDR) and HR outcomes indirectly [51].

Cohesin provides sister chromatid cohesion by loading during replication. However, when DSB occurs, de novo cohesin molecule loading is observed, providing the so-called damage-induced cohesion (DI cohesion) [140,141]. DI cohesion also occurs after replication in G2/M [132]. Post-translational modifications of the components of the cohesin complex play an important role in maintaining the stability of cohesin molecules providing DI cohesion. It is pertinent to note that DI cohesion occurs not only in the DSB region, but also throughout the genome [142,143]. Apparently, this is important for preventing the separation of unrepaired chromatids. Details on DI cohesion and the role of cohesin in it can be found in a comprehensive review [144].

Discovery of LE activity created a new viewpoint on cohesin’s role in DNA repair. A combination of new genome-wide and site-specific methods, such as Hi-C, ChIP-seq, and scheduled induction of DSBs [145,146], has allowed observation of DNA folding around the break with high resolution and in real time. In addition, inducible degron systems have brought unprecedented efficiency and control over protein depletion [147], which is important, since even low residual levels of cohesin might sustain physiological DSB repair.

Recent work based on these methods described how LE activity is exploited by cells to establish γH2AX signaling over large territories [51]. Phosphorylation of histone H2AX on Ser139 (γH2AX) is a central aggregator of DNA damage response [148,149]. Prior reports presented evidence that γH2AX signaling around the DSBs spreads linearly over megabase-scale distances, potentially overlapping with local TADs [150,151,152]. The speed of γH2AX spreading was found to be similar to LE speed of the cohesin complex (0.6–2.5 kb/s) calculated in different systems [28,56,146,153], which hinted that cohesin might facilitate the process. To verify this hypothesis, Arnould and colleagues monitored scheduled DSBs induced by AsiSI or Cas9 nucleases in human cells. They discovered that cohesin was present at the break sites together with the early DSB sensors, MRN and ATM kinase, and was required for gradual spreading of γH2AX over the local TAD. Averaged Hi-C maps for the DSB regions displayed characteristic ‘stripe’ patterns similar to the one-sided loops [33,46]. This led to a conclusion that cohesin complexes anchored at DSBs act as ‘conveyer belts’ to present chromatin for ATM which is bound only at the vicinity of the DNA ends. Such DSB-anchored one-sided loop arrest is an efficient way to utilize LE activity for DDR signal amplification and constraining DNA repair within a specifically decorated TAD. Importantly, while ATM inhibition completely abolished γH2AX signaling, depletion of Rad21 only slightly affected the γH2AX levels (ca. 10% decrease) [51]. Future research could be aimed at identifying a potential kleisin subunit that governs this function at the DSB.

Another seminal paper provided a new insight into the local interactions of the DSB ends [136]. Using yeast recombination reporters combined with Hi-C, the authors monitored contact frequencies after DSB induction and repair in G2/M arrested cells. They discovered the enrichment of DNA contacts locally around the break in a 10–25 kbp region (‘local interaction pattern’ (LIP)). Formation of this pattern was cohesin independent. Careful examination of the contributing DNA repair factors revealed that LIP relied on resection (mre11, exo1) and 9-1-1 complex, the DNA damage checkpoint clamp [154], while Rad51 and Rad52 post-resection factors were irrelevant for LIP topological changes [136]. Collectively, their data show a process in which 9-1-1 tethers resected DSB ends together, replacing initial MRN binding, which causes local DNA folding. At the same time, Hi-C analysis of DSB sites on a larger genomic scale revealed that cohesin LE activity creates one-sided loops anchored at the DSBs, similar to stripe patterns from another article [51]. Interactions of the DSB ends were limited to the side-specific cis contacts. Thus, LE limits the mobility of the DNA ends to a special isolated topological domain to avoid ectopic recombination.

In addition to these base DSBR orchestrating functions, LE activity has also been implemented in DSBR promotion in specialized biological processes, such as bringing DNA segments together during V(D)J recombination [155], establishing meiotic crossover formation [156], and topoisomerase-induced DNA decatenation [157,158].

Discovery of the fundamental role of LE activity in DNA repair raises many exciting questions for future studies. One of the central problems is the difference between de novo loaded and run-on cohesin molecules at the DSB and how they cooperate with the DNA repair foci proteins. What anchors cohesin at the DSB for LE: ATM [51], 9-1-1 [136], SMC5/6 [125], or other unidentified factors? What is the purpose of global induction of cohesin binding and how are stoichiometry and dynamics of cohesin molecular exchanges regulated by DDR at chromosomal, TAD, and local levels [51,133,159]? How does cohesin extrude chromatin in ‘difficult’ chromatin contexts (heterochromatin) to manage DSB repair [134,160]? How does cohesin interact with different types of DSBs, such as collapsed replication forks, topoisomerase-induced damage, or meiotic DSBs?

### 4.4. Loop Extrusion for Chromatin Topology

The linear sizes of the DNA molecule significantly exceed the linear size of the nucleus, therefore formation of the DNA loops is essential to fit the genome into the nuclear space. Here, we describe how loop extrusion compacts the genome and defines the shape and physical properties of interphase and metaphase chromosomes.

#### 4.4.1. Formation of Mitotic Chromosomes by Loop Extrusion

It is well known that condensins are essential for mitotic compaction of chromosomes. Recently, a model was proposed where condensin I and II pack DNA into arrays of consecutive loops condensed around a central axis [161]. Either condensin can mediate loop array formation. However, condensin II is required for the helical twisting of the scaffold from which loops emanate, whereas condensin I modulates the size and arrangement of nested inner loops. According to this model, condensin I primarily decreases the width of the chromosome, whereas condensin II drives lengthwise compaction. Thus, condensins are pivotal factors determining the shape of mitotic chromosomes.

Interestingly, DNA compaction during mitosis also affects the shape of chromosomes in the following interphase. This was recently shown by comparing eukaryotic species with and without condensin II subunits. Those species lacking condensin II subunits display lower lengthwise compaction of chromosomes during mitosis. Lower lengthwise compaction allows intermingling between chromosomes and establishment of the interactions between HP1-rich centromeres [162]. This causes centromere clustering [20] after mitosis, resulting in the so-called Rabl-like configuration of the nucleus, where centromeres are co-localized at the nuclear periphery and chromosomes acquire elongated shapes [21].

In addition to centromere clustering, the elongated shape of mitotic chromosome results in the non-spherical shape of chromosome territory during the next interphase, because chromosome relaxation after mitosis starts from this specific conformation [163]. These results show how condensin action during mitosis indirectly affects nuclear organization in the following interphase.

#### 4.4.2. Shaping Interphase Chromosomes by Loop Extrusion

In Drosophila, condensin not only impacts the shape of the interphase chromosomes indirectly, but also binds chromatin after mitosis to directly influence chromosome territory localization. Condensin regulates alignment of homologous chromosome territories, in a phenomenon called homologous chromosome pairing. The mechanism of homologous chromosome pairing is not fully understood, and it is not clear whether loop extrusion *per se* is important for this process. However, it is known that condensin activity counteracts pairing, and its depletion increases frequencies of contacts between homologous chromosomes [164,165]. Cohesin also binds chromatin during interphase in Drosophila cells, and its activity increases gene looping and formation of active compartmental domains [166]. Interestingly, knockdowns of condensin and cohesin show opposite effects on chromatin contacts in Drosophila, therefore these SMC complexes counteract each other during interphase.

In vertebrates, the primary effects of condensin on chromatin folding during interphase remain elusive. Although condensin II has nuclear localization throughout the cell cycle, acute depletion does not significantly affect nuclear architecture [20]. Moreover, although localized in the nucleus, condensin II probably does not translocate chromatin during interphase [167].

Another loop-extruding complex, cohesin, binds interphase chromatin and forms loops during the interphase. In addition to the role in DNA repair and gene expression regulation described in other sections of this review, cohesin-mediated looping is important to maintain chromosome territories. Physical simulations show that formation of loops is required to maintain lengthwise compaction of chromosomes, which establishes chromosome territories and suppresses interchromosome entanglements [168,169]. Moreover, extrusion decreases chromosome mobility, and cohesin and CTCF constrain the dynamics of sequences in *cis* interactions [57]. In addition, cohesin directs topoisomerase II activity to disentangle long-range compartment interactions. This evidence supports the hypothesis that cohesin contributes to formation and maintenance of chromosome territories in vertebrate cells.

On the other hand, a recent experiment shows maintenance of a territorial organization of interphase chromosomes in pre-mitotic, cohesin-depleted human HCT116 cells [170]. Interestingly, cells without cohesin can pass through endomitosis, producing a multilobulated nucleus in daughter cells. Within this multilobulated nucleus, chromosome territories are reconstituted in the absence of cohesin. Other global features of higher-order chromatin organization were restored after endomitosis in cohesin-depleted cells as well. These results make the role of cohesin in establishment and maintenance of chromosome territories questionable, asking for further studies on different experimental systems.

## 5. Conclusions

The loop extrusion process plays an essential role in different genomic processes. With the advent of novel biochemical, microscopy, and computational technologies exploring genome architecture, we are gaining more details about the complex biology of this process. Yet, many details are still unknown. Why do some mutations affecting genome architecture cause disease whereas others do not? When and how did CTCF gain the ability to interfere with the loop extrusion process? What are other barriers limiting loop extrusion, and what is their role in genome regulation? We believe that further methodological developments of chromosome conformation methods and complementary techniques [171,172,173,174,175,176], including single-cell protocols [177], will help to answer these questions.

## Data Availability

Not applicable.
